# Response of the ductus arteriosus to acetaminophen or indomethacin in extremely low birth weight infants

**DOI:** 10.1038/s41372-024-02199-5

**Published:** 2024-12-18

**Authors:** Courtney C. Sutton, James C. Slaughter, Mhd Wael Alrifai, Jennifer Hale, Jeff Reese

**Affiliations:** 1 Department of Pharmacy, Monroe Carell Jr. Children’s Hospital at Vanderbilt, Nashville, TN 37232 USA; 2 Department of Biostatistics, Vanderbilt University Medical Center, Nashville, TN USA; 3 Division of Neonatology, Department of Pediatrics, Vanderbilt University Medical Center, Nashville, TN 37272 USA; 4 Department of Biomedical Informatics, Vanderbilt University Medical Center, Nashville, TN 37272 USA

**Keywords:** Congenital heart defects, Paediatrics, Drug therapy

## Abstract

**Objective:**

Acetaminophen and indomethacin are used for medical management of a patent ductus arteriosus. This study compared the efficacy of these agents in ELBW infants.

**Study design:**

This was a retrospective study of all courses of indomethacin and acetaminophen. Baseline characteristics, details of pharmacologic therapy, toxicity, and acetaminophen serum concentrations were collected. The primary analysis compared rates of ductus closure with indomethacin versus acetaminophen using Pearson’s Chi-squared test.

**Results:**

Ductus closure after a single course of therapy was similar between acetaminophen and indomethacin 16% vs. 18%, (*p* = 0.79). No differences were found in gestational age, birth weight, patient acuity, toxicity, or acetaminophen concentrations between those courses that resulted in closure compared to those that did not. When comparing single-agent exposure, indomethacin was initiated earlier (9.0 vs. 13.5 days, *p* = 0.022) but PDA closure rates were similar between groups.

**Conclusion:**

Acetaminophen and indomethacin produced similar rates of ductus closure in this population.

## Introduction

Extremely low birth weight (ELBW) infants are at risk for various complications related to preterm birth [1]. One of these frequent complications is a persistently patent ductus arteriosus (PDA) [2]. The ductus arteriosus (DA) is a necessary part of fetal circulation, but generally closes spontaneously after birth in term infants [2]. In preterm infants born at 27-28 weeks gestation, the DA remains patent in 60-70% of cases at day of life 7, with 87% of infants born at 24 weeks gestation suffering from a PDA [3]. The presence of a hemodynamically significant PDA can lead to further short-term complications, such as respiratory compromise, renal failure, necrotizing enterocolitis (NEC), and long-term complications, including the development of bronchopulmonary dysplasia (BPD) [2, 4]. Historically, up to 70% of infants with a PDA have required medical or surgical closure [4]. Surgical ligation has been associated with various immediate and long-term complications, including post-ligation cardiac syndrome, phrenic nerve injury and vocal cord paralysis, and infection, as well as increased rates of retinopathy of prematurity, chronic lung disease, and neurodevelopmental impairment [5, 6] Additionally, the small size of these infants may limit their eligibility for surgical management [6]. Given these barriers, a trial of pharmacologic closure is generally considered prior to proceeding with surgical or device closure in the ELBW population [5].

The non-steroidal anti-inflammatory drugs (NSAID) indomethacin and ibuprofen have been the mainstay of therapy for pharmacologic closure of a PDA for over 40 years [4, 7, 8]. Side effects of NSAID use include renal dysfunction, gastrointestinal (GI) bleeding, GI perforation, and platelet dysfunction, which limits their use in ELBW infants [4, 9]. Over the past decade, data has emerged suggesting acetaminophen is also effective at achieving ductus closure, with a more desirable short-term side effect profile, including lower rates of GI bleeding, lower serum creatinine levels, higher platelet count and higher daily urine output as compared to traditional NSAID agents [10–15]. Although indomethacin is considered a more potent inhibitor of prostaglandin synthesis, several studies have reported that acetaminophen is equally efficacious to ibuprofen and indomethacin for ductus closure in preterm infants [12, 14–21]. However, when looking specifically in preterm infants <28 weeks, the evidence regarding closure efficacy is conflicting [12, 21–25].

At our institution, the decision of which agent to use for medical closure is at the discretion of the attending provider, with dosing and monitoring guidelines available for both indomethacin and acetaminophen. We hypothesized that PDA closure would be more successful with indomethacin than acetaminophen. Thus, the objective of this study was to compare the efficacy of pharmacologic agents for ductus closure in patients <1000 g at birth in our neonatal intensive care unit (NICU).

## Methods

### Patient population

This was a retrospective cohort study of all ELBW infants admitted to the Monroe Carell Jr. Children’s Hospital at Vanderbilt NICU who received either indomethacin or acetaminophen for ductus closure between November 2017 and March 2021. Pharmacy administration records were used to identify all patients who received at least one dose of indomethacin or acetaminophen during the study period. Infants that received only prophylactic indomethacin or those who received acetaminophen for another indication were excluded from the study. At this institution, a single dose of prophylactic indomethacin (0.2 mg/kg) is routinely given at 12 hours of age to all infants <1000 g birthweight unless contraindicated by recent maternal indomethacin tocolysis, thrombocytopenia, acute kidney injury (AKI), or intraventricular hemorrhage [26]. Institutional guidelines for routine PDA screening in the first week of life were not in place at the time of this study, so all echocardiograms were obtained using a pragmatic approach in response to clinical suspicion of a PDA. A PDA was considered hemodynamically significant (hsPDA) by echocardiography if pediatric cardiologists considered the ductus vessel size to be medium or large by color Doppler with continuous left-to-right shunt plus increased left atrial (LA) size (LA/aorta ratio >1.5) or retrograde diastolic aortic flow at the level of the diaphragm. Hemodynamically significant PDAs were managed per protocol with modest fluid restriction (120–130 mL/kg/d) and maintenance of positive end-expiratory pressure of at least 5 cmH_2_O [26].

### Pharmacologic therapy

While no formal guidance encouraged the use of one agent over another during the study period, institutional guidelines were available for each medication. Intravenous (IV) indomethacin was administered at 0.2 mg/kg/dose over 30 min every 12 h for 3 doses. If clinical signs of a PDA were still present or the patient was high risk, defined as extreme prematurity or lack of improvement in respiratory status despite treatment, a 4th dose was administered 24 h after the 3rd dose. Both IV and enteral acetaminophen were administered as 15 mg/kg/dose every 6 h for 3 days but could be extended if the PDA was present after the initial 3-day course. Intravenous acetaminophen was administered over 15 min. Additionally, the dose could be increased to 20 mg/kg/dose every 6 h at the discretion of the attending physician. Acetaminophen serum concentrations, aspartate aminotransferase (AST) and alanine aminotransferase (ALT) were drawn prior to the 9th dose of acetaminophen. If the hsPDA persisted after the initial course of medication, the course could be repeated, or the alternative pharmacologic agent could be trialed at the discretion of the attending neonatologist. For those unresponsive to medical management, pediatric cardiology was consulted for consideration of ligation or placement of an occlusion device (MVP™-5Q, Medtronic, Minneapolis, MN or Amplatzer Piccolo™ Occluder, Abbott, Santa Clara, CA).

### Data collection

Demographic data collected from the electronic medical record included gestational age at birth, birth weight, birth hospital location, method of delivery, race, sex, receipt of indomethacin prophylaxis, Apgar scores, and a diagnosis of intrauterine growth restriction (IUGR) on the patient’s problem list. Patients were not excluded if they received more than one agent or course; however, demographic information was only included for the patient once per drug. Age at treatment, duration of therapy, medication selection, medication dose, and route of administration were collected for each medication treatment course. Patients that were transitioned between intravenous and enteral acetaminophen were counted in both administration routes. Respiratory support (modality, positive end expiratory pressure, mean airway pressure), fraction of inspired oxygen, and vasopressor requirement on the day of treatment initiation were also recorded. Respiratory support data were used to calculate a respiratory severity score (RSS) for infants on mechanical ventilation (RSS_MV_) or continuous positive airway pressure (RSS_CPAP_). Vasopressor agent and dose were used to calculate a vasoactive-inotropic score (VIS) as follows: dopamine dose (μg/kg/min) + dobutamine dose (μg/kg/min) + 100 x epinephrine dose (μg/kg/min) + (10 x milrinone dose (μg/kg/min)) + 10 x vasopressin dose (mU/kg/min) + 100 x norepinephrine dose (μg/kg/min) [27]. The VIS^max^ was the maximum VIS score on the day of PDA treatment initiation. Additionally, acetaminophen serum concentrations, AST, and ALT during acetaminophen treatment were collected as markers of toxicity related to therapy. Elevations in AST or ALT were noted if they were above the reference range for infants. Likewise, if patients in the indomethacin group required doses to be held, the investigators noted if suspected AKI or thrombocytopenia were present. The final disposition of the DA was also collected to determine if additional pharmacologic therapy or surgical intervention was needed. Patients could receive multiple courses of therapy for PDA closure, defined as a new start of a different pharmacologic agent or a new start of the same medication therapy at least 48 h after discontinuation of the previous course.

### Outcomes

The primary outcome of the study was ductus closure confirmed by echocardiogram. We elected to evaluate outcomes by drug course rather than by patient number to better reflect the response to each pharmacologic agent. Secondary outcomes included evaluating the relationship of acetaminophen serum concentrations and ductus closure, safety of each agent, association of baseline characteristics and PDA closure, and rates of required surgical closure with each medication.

### Statistical analysis

Demographic information and other continuous variables were summarized by type of drug used or PDA closure in separate tables. The Mann-Whitney U was used to compare continuous variables between groups and Pearson’s Chi-squared test was used to compare categorical variables between groups. Race was collapsed to three levels (White, African American, and Other) for statistical tests while all race categories are shown in the tables. The mean difference in acetaminophen levels by ductus open or closed is shown using a Gardner-Altman estimation plot. A 95% confidence interval for the mean difference was estimated using a bias corrected and accelerate Bootstrap sample with 5000 replications. Sensitivity analyses include multivariable logistic regression where we estimated effect of type of drug administered controlling for the potential confounding effects of gestational age (or birth weight), sex, and age at drug start. Due to the limited number of events, separate adjusted models were considered for each potential confounder. The study was powered to detect an 18% difference between acetaminophen and indomethacin groups at a significance level of 0.05 based on enrolling 45 subjects in one group and 90 subjects in another group. Statistical analyses were performed using R version 4.4.1. This study was approved by the Vanderbilt University Institutional Review Board.

## Results

### Patient population

Ninety-two courses of acetaminophen and 44 courses of indomethacin were administered to 97 patients. Twenty-two patients received both acetaminophen and indomethacin therapy. Gestational age, birth weight, sex, race, mode of delivery, delivery location, presence of IUGR, and rates of indomethacin prophylaxis were similar between the acetaminophen and indomethacin treated groups (Table 1). One-minute and five-minute Apgar scores were lower in the acetaminophen-treated group (2.0 vs. 4.5, *p* = 0.007 and 6.0 vs. 7.0, *p* = 0.025).

**Table 1:** Patient characteristics by first course.

	Acetaminophen (*n* = 71)	Indomethacin (*n* = 26)	*p* value
GA at birth, weeks (median, IQR)	25 [24–26]	26 [24–27]	0.2
Birth weight, grams (median, IQR)	670 [585-874]	792 [655-840]	0.24
Female	43 (61%)	18 (69%)	0.43
Cesarean delivery	59 (83%)	22 (85%)	0.86
IUGR present	20 (28%)	7 (27%)	0.9
Inborn delivery	47 (66%)	22 (85%)	0.076
Race			
African American	22 (31%)	9 (35%)	0.39
African American/White	2 (3%)	0 (0%)	
Asian	1 (3%)	2 (8%)	
Asian/White	2 (3%)	0 (0%)	
Hispanic/Latino	0 (0%)	1 (4%)	
Unknown	6 (8%)	0 (0%)	
White	36 (51%)	14 (54%)	
Apgar score (median, IQR)			
1 min	2.0 [1.0-4.5]	4.5 [3.0–6.0]	0.001
5 min	6.0 [4.0-7.0]	7.0 [5.2–8.0]	0.046
10 min	7.0 [6.0-8.0]	7.0 [6.0–7.0]	0.41
Received indomethacin prophylaxis	43 (61%)	17 (65%)	0.67

### Toxicity

Elevations in liver function tests were seen in 8 courses (9%) in the acetaminophen treatment group. Five of these patients were dependent on parenteral nutrition with suspected parenteral nutrition cholestasis prior to starting acetaminophen. Elevations in liver enzymes were transient in the remaining 3 patients. One patient (2%) required indomethacin doses to be held secondary to a transiently elevated serum creatinine and decreased urine output (Table 2).

**Table 2:** Results by treatment course.

	Acetaminophen (*n* = 92)	Indomethacin (*n* = 44)	*p* value
Age at therapy start, days (median, IQR)	13.0 [8.0–19.2]	12.0 [7.5–16.8]	0.3
Vasopressor requirement^a^, *n*	8 (9%)	3 (7%)	0.71
VIS^MAX^ (median, IQR)	5.5 [5–10]	5 [5–6]	---
Respiratory support type^a^, *n*			
Mechanical ventilation	59	23	---
CPAP	31	21	
High flow nasal cannula	2	0	
RSS_MV_^a,b^ (median, IQR)	3.5 [2.8–4.8]	3.8 [2.8–5.1]	0.79
RSS_CPAP_^a,c^ (median, IQR)	1.4 [1.1–1.6]	1.4 [1.3–1.5]	0.54
Toxicity^d^	8 (9%)	1 (2%)	0.16
Successful PDA closure	15 (16%)	8 (18%)	0.79

^a^On day of PDA therapy start.

^b^RSS_MV_ = (median FiO2 x median MAP)/100.

^c^RSS_CPAP_ = (median PEEP x median FiO2)/100.

^d^Toxicity in acetaminophen group defined as elevated AST or ALT above reference range for age; toxicity in indomethacin group defined as suspected acute kidney injury or thrombocytopenia requiring a change in therapy.

### Efficacy

Ductus closure after a single course of therapy was similar in the acetaminophen and indomethacin treated groups (16% vs. 18%, *p* = 0.79). In separate multivariable models where we controlled for the potential confounding effect of either gestational age, birth weight, sex, or age at drug start, there was also no evidence of an association between type of therapy and the odds of ductus closure. When comparing courses with successful closure and those without, no differences were seen in baseline characteristics, patient acuity or medication use patterns (Table 3).

**Table 3:** Patient characteristics by courses with and without ductus closure.

	PDA Closed (*n* = 23)	PDA Not Closed (*n* = 113)	*p* value
GA at birth, weeks (median, IQR)	25 [24–26]	25 [24–26]	0.83
Birth weight, grams (median, IQR)	730 [612–855]	710 [590–870]	0.9
Female	13 (57%)	76 (67%)	0.32
Cesarean delivery	20 (87%)	92 (81%)	0.53
IUGR present	8 (35%)	33 (29%)	0.59
Inborn delivery	16 (70%)	81 (72%)	0.84
Race			
African American	7 (30%)	38 (34%)	0.45
African American/White	1 (4%)	2 (2%)	
Asian	1 (4%)	7 (6%)	
Asian/White	1 (4%)	1 (1%)	
Hispanic/Latin	0 (0%)	1 (1%)	
Hispanic/Latino	1 (4%)	0 (0%)	
Unknown	1 (4%)	6 (5%)	
White	11 (48%)	58 (51%)	
Apgar score (median, IQR)			
1 min	2 [1–6]	3 [1–6]	0.84
5 min	7 [5–7]	6.5 [4.0–8.0]	0.77
10 min	7.0 [6.2–7.8]	7.0 [6.0–7.0]	0.71
Vasopressor requirement^a^, *n*	4 (17%)	7 (6%)	0.073
VIS^MAX^ (median, IQR)	5.5 [5-6.25]	5 [5–10]	---
Respiratory support type^a^, *n*			---
Mechanical ventilation	13	69	
CPAP	10	42	
High flow nasal cannula	0	2	
RSS_MV_^a,b^ (median, IQR)	3.7 [2.9–5.3]	3.4 [2.7–5.0]	0.42
RSS_CPAP_^a,c^ (median, IQR)	1.3 [1.1–1.4]	1.4 [1.1–1.6]	0.41
Intravenous Acetaminophen	11/15 (73%)	60/77 (78%)	0.7
Enteral Acetaminophen	8/15 (53%)	25/77 (32%)	0.12
Age at therapy start, days (median, IQR)	12 [7.5–16.5]	13 [8.0–19.0]	0.58
Age < 14 days at start^a^	13 (57%)	60 (53%)	0.76

^a^On day of PDA therapy start.

^b^RSS_MV_ = (median FiO2 x median MAP)/100.

^c^RSS_CPAP_ = (median PEEP x median FiO2)/100.

### Acetaminophen treatment

Acetaminophen was used more often than indomethacin during the study period with documented ductus closure on echocardiogram noted in 16% of cases. There was no difference in PDA closure based on the route of acetaminophen administration (Table 3). Of the courses that did not result in ductus closure, 15 courses were followed by additional medical management (either a repeat course of acetaminophen or a course of indomethacin).

### Acetaminophen serum concentrations

**Fig. 1:**
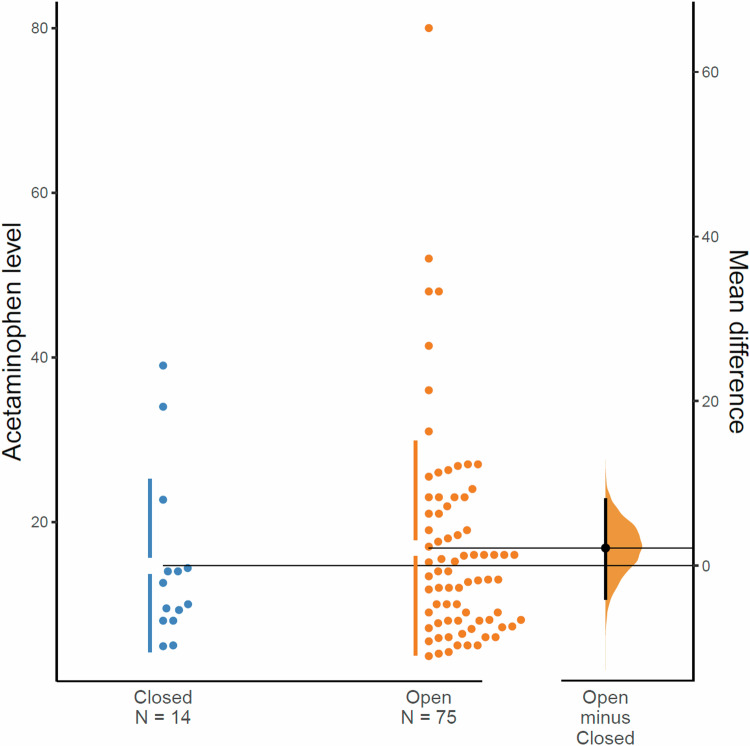
Acetaminophen level with and without ductus closure.

Acetaminophen serum concentrations ranged from <3 mcg/mL to 80 mcg/mL. Serum concentrations did not differ in the group with successful closure compared to those who did not close with acetaminophen (11.3 vs. 14.0, *p* = 0.47) (Fig. 1).

### Indomethacin treatment

Indomethacin resulted in successful closure in 18% of courses. When the PDA persisted, 12 courses were followed with additional medical management (either a repeat course of indomethacin or a course of acetaminophen).

### Acetaminophen vs. indomethacin

Patients who received only acetaminophen or only indomethacin for pharmacologic closure were also analyzed. Sixty-six courses of acetaminophen were administered to 58 patients, and 18 courses of indomethacin were administered to 16 patients. No significant differences were noted in baseline characteristics or patient acuity between the two groups except for lower one-minute and five-minute Apgar scores in the acetaminophen group (Table 4). The median age at start of therapy was lower in the indomethacin-treated group (9 days vs. 13.5 days, *p* = 0.022), although further analyses showed no difference in the effect of medication on closure rates when controlling for age at start or evidence of effect modification by age at start on drug efficacy. When adjusting for age at start, the adjusted odds ratio for indomethacin vs. acetaminophen is 1.11. There was no difference in rate of confirmed PDA closure (28% vs. 21%, *p* = 0.56). Of those patients that did not close with medical therapy, surgical intervention was lower in the indomethacin treated group, though not statistically significant (11.5% vs. 36%, *p* = 0.069).

**Table 4:** Patients receiving single pharmacologic therapy.

	Acetaminophen (*n* = 58)	Indomethacin (*n* = 16)	*p* value
GA at birth, weeks (median, IQR)	25 [24–26]	26 [24–27]	0.37
Birth weight, grams (median, IQR)	685 [600–877]	808 [648–872]	0.36
Female	35 (60%)	10 (62%)	0.88
Cesarean delivery	48 (83%)	14 (88%)	0.65
IUGR Present	16 (28%)	5 (31%)	0.77
Inborn delivery	38 (66%)	15 (94%)	0.027
Race			
African American	19 (33%)	4 (25%)	0.21
African American/White	2 (3%)	0 (0%)	
Asian	0 (0%)	1 (6%)	
Asian/White	2 (3%)	0 (0%)	
Hispanic/Latino	1 (2%)	1 (6%)	
Unknown	5 (9%)	0 (0%)	
White	29 (50%)	10 (62%)	
Apgar score			
1 min	2.0 [1.0–4.0]	4.5 [3.0–6.0]	0.004
5 min	6.0 [4.0–7.8]	7.0 [6.0–8.0]	0.028
10 min	7.0 [6.0–8.0]	7.0 [7.0–7.0]	0.92
Received indomethacin prophylaxis	34 (59%)	10 (62%)	0.78
Vasopressor requirement^a,b^, *n*	8 (12%)	3 (17%)	0.61
VIS^MAX^	5.5 [5.0–10.0]	5.0 [5.0–6.0]	---
Respiratory support type^a,b^, *n*			---
Mechanical ventilation	44	8	
CPAP	20	10	
High flow nasal cannula	2	0	
RSS_MV_^a,b,c^	3.5 [2.9–5.2]	4.3 [3.5–5.3]	0.53
RSS_CPAP_^a,b,d^	1.4 [1.1–1.5]	1.4 [1.2–1.5]	0.86
Age at therapy start, days^a,b^ (median, IQR)	13.5 [9.0–19.0]	9.0 [5.2–15.0]	0.022
Age < 14 days at start^a,b^	33 (50%)	12 (67%)	0.21
Toxicity^b,e^	8 (12%)	1 (6%)	0.42
Successful PDA closure^b^	14 (21%)	5 (28%)	0.56
Surgical closure	21 (36%)	2 (11.5%)	0.069

^a^On day of PDA therapy start.

^b^Data presented by course: 66 acetaminophen courses, 18 indomethacin courses.

^c^RSS_MV_ = (median FiO2 x median MAP)/100.

^d^RSS_CPAP_ = (median PEEP x median FiO2)/100.

^e^Toxicity in acetaminophen group defined as elevated AST or ALT; toxicity in indomethacin group defined as suspected acute kidney injury or thrombocytopenia requiring a change in therapy.

## Discussion

In this study of <1000 g birth weight infants, there was no difference in successful PDA closure with the use of acetaminophen or indomethacin. While this has been shown in the general preterm population, this study adds to the literature for the ELBW population where current data is conflicting [12, 14–25]. El-Mashad and colleagues compared paracetamol, ibuprofen, and indomethacin for PDA closure in infants <28 weeks of age and found that paracetamol was as effective as the NSAID agents with fewer noted side effects [12]. In contrast, Dani and colleagues found a higher rate of failure with first cycle acetaminophen as compared to ibuprofen in those born <28 weeks gestation, with a higher failure rate noted in the 22–23 week group [22]. Similarly, the PDA-TOLERATE secondary analysis found indomethacin to be more effective for PDA constriction in those <28 weeks than acetaminophen [21]. It is important to note that dosing in the PDA-TOLERATE trial group was lower than the acetaminophen dosing used in the El-Mashad report and in our study group, which may have impacted their outcomes [12, 21]. Currently published literature describes various acetaminophen dosing regimens used with success in the neonatal population, with evidence to suggest that extremely preterm neonates may require higher doses to achieve ductus closure than older preterm infants [9, 23]. Further studies to define the optimal dose and duration of acetaminophen treatment, particularly in this population, are needed.

The rate of PDA closure in this study was low for both medications compared to previous reports in the literature. Acetaminophen has been reported to successfully close the PDA in 6 to 80% of patients, whereas indomethacin has shown success in 32 to 81% of patients, depending on gestational maturity [7, 9, 12, 16, 21, 23, 28–31]. While some authors have considered success with an agent to be closure, others have considered significant constriction of the PDA as a successful outcome. When considering both confirmed closure on echocardiogram and constriction of the PDA as a successful outcome, indomethacin appeared to be more efficacious in our population, with 87.5% of infants requiring no further intervention, compared to 67% in the acetaminophen treated group, which corresponds to the lower surgical intervention rates seen in the indomethacin group. The indomethacin-treated patients were initiated on therapy earlier than the acetaminophen-treated group, which we suspected may impact the efficacy of therapy, although no evidence of effect modification existed in additional analyses. Larger prospective studies are needed to confirm these findings.

Acetaminophen concentrations were found to have no association with ductus closure. These findings are consistent with previous reports in the literature [7, 21, 29]. Given this information and the low likelihood of hepatotoxic metabolite generation due to immature metabolic pathways in the neonate, we no longer obtain acetaminophen serum concentrations when treating for PDA [9, 32].

As evidenced by the present data, providers at our institution give preference to acetaminophen over indomethacin when medical treatment is required. This is often to avoid the adverse effects of indomethacin. We observed few side effects with the use of indomethacin in this study; however, the study was not designed to assess drug safety and the population that received indomethacin was small. Furthermore, those infants more likely to experience side effects related to NSAIDs (pre-existing thrombocytopenia, renal dysfunction, hemodynamic instability, etc.) were likely selected out for acetaminophen therapy instead. Although the side effect profiles of these agents differ, the overall rate of short-term side effects in our study were similar between the two agents.

Multiple studies have assessed efficacy for PDA closure and remarked on the short-term safety profile of acetaminophen in the preterm population, but long-term safety data in the developing infant is lacking. Studies performed in murine models have indicated that exposure to acetaminophen during periods of critical brain development may produce lasting effects on cognitive function, even into adulthood [33]. In humans, maternal acetaminophen use during pregnancy may impact neurodevelopment, noting associations with delayed milestone achievement, delayed language development, and autism, with early childhood exposure also associated with the development of autism [34]. Finally, while the risk of hepatotoxicity in this population seems low, owing to the underdevelopment of CYP2E1 expression in the liver, CYP2E1 may have earlier expression in the human lung, making the lungs a potential target for toxicity with acetaminophen exposure [35]. Murine lung models in the early alveolar development stage have shown susceptibility to injury following acetaminophen exposure, with patterns of injury similar to that of preterm infants with BPD [35]. As suggested by others, the long-term safety of acetaminophen exposure deserves further study.

### Limitations

This study is limited by its retrospective design and reliance on accurate documentation in the electronic medical record. There was also no way to standardize which infants received a particular medication based on patient factors, nor was the documentation clear regarding why one agent was selected over another. In those infants who received no further medical or surgical intervention, it is unclear if this was due to adequate ductus constriction or if it was due to the inability to tolerate further medical or surgical management. This study looked at the short-term safety profile of both acetaminophen and indomethacin but did not look at longer-term safety outcomes such as necrotizing enterocolitis or GI bleeding. While this study was powered to detect an 18% difference, a larger sample size may have allowed for smaller detections in therapeutic efficacy.

## Conclusions

There was no difference in the rate of successful PDA treatment in ELBW infants with the use of acetaminophen or indomethacin for pharmacologic closure; however, the overall rates of successful closure with medical management were low in this population.

## Data Availability

All data generated or analyzed during this study are included in this published article.
